# Exploring the Interactions Between Epidural Analgesia, Extubation and Reintubation Outcomes in Infants in Neonatal Care Units: A Retrospective Cohort Study

**DOI:** 10.3390/children12030275

**Published:** 2025-02-24

**Authors:** Mihaela Visoiu, Stephanie Parry, Tyler H. Augi, Danielle R. Lavage, Scott E. Licata, Holly A. Turula, Doreen E. Soliman

**Affiliations:** 1 Department of Anesthesiology and Perioperative Medicine, University of Pittsburgh School of Medicine, UPMC Children’s Hospital of Pittsburgh, Pittsburgh, PA 15261, USA; licatase@chp.edu (S.E.L.); solimande@upmc.edu (D.E.S.); 2 Department of Anesthesiology and Perioperative Medicine, Department of Pediatrics, Division of Newborn Medicine, UPMC Children’s Hospital of Pittsburgh, Pittsburgh, PA 15261, USA; parrysm@upmc.edu; 3 University of Pittsburgh School of Medicine, Pittsburgh, PA 15261, USA; tha25@pitt.edu; 4 Department of Anesthesiology and Perioperative Medicine, University of Pittsburgh School of Medicine, Pittsburgh, PA 15261, USA; drl49@pitt.edu; 5 Department of Anesthesiology and Perioperative Medicine, UPMC Children’s Hospital of Pittsburg and Shadyside Hospital, Pittsburgh, PA 15261, USA; turulaha@upmc.edu

**Keywords:** neonates, epidural catheter, morphine, pain scores, extubation, reintubation, sedation scores

## Abstract

Background/Objectives: Continuous epidural analgesia is desirable for improving infant outcomes after surgeries. However, its contribution to facilitating extubation is not well known. Methods: A retrospective chart review was conducted at the UPMC Children’s Hospital of Pittsburgh to identify all infants who received an epidural catheter between 2018 and 2024 and required postsurgical admission to the Neonatal Intensive Care Unit (NICU). The study examined the timing of extubation and reintubation, along with associated factors, in 100 infants who underwent major surgeries. Results: In total, 100 infants, 43 females and 57 males, 40 (38.39–42.07) weeks corrected gestational age, 3 (2.52–3.42) kg received epidural catheters. Sixty-two patients had a pulmonary condition. Of 45 infants extubated in the operating room, 32 received fentanyl intraoperatively, and 16 required a morphine infusion in the NICU. Among 55 infants that remained intubated, 24% underwent a thoracic procedure, 46 received intraoperatively fentanyl, and 21 needed an opioid infusion postoperatively. The extubation day was median (IQR) 2 (1–4), and 24% remained intubated beyond day 5. Twelve infants were intubated preoperatively, and six required prolonged ventilation beyond day 5. Of 15 infants that required reintubation, 8 received a morphine infusion. The medians (IQR) of the average of three pain and sedation scores before reintubation were 1.67 (1–3) and 0 (−1.67–0), respectively. Conclusions: Epidural analgesia may facilitate early extubation in some infants undergoing surgeries. Morphine infusion was administered at a similar rate between infants extubated and those who remained intubated, and its role in delaying extubation timing remains unclear.

## 1. Introduction

In the rapidly advancing field of neonatal care, infants are exposed to various types of painful procedures, including surgeries [1,2]. One critical aspect of neonates’ perioperative care is the timing of extubation, as early or delayed extubation can significantly impact their recovery and overall outcomes. Various factors, such as preoperative intubation status, type of surgical procedure, presence of an undelying pulmonary condition, type of postoperative analgesia, such as opioids and continuous epidural analgesia (CEA), pain scores, and sedation, can influence the timing and success of extubation.

Opiates, especially morphine, are the cornerstone for postoperative pain control after surgery in neonates [3,4,5]. However, morphine administration is associated with undesirable side effects, such as cardiorespiratory complications [6], medication-related adverse events [6], tolerance [7], concerns about long-term developmental delays [8,9], and delayed extubation. These challenges make continuous epidural analgesia (CEA) a desirable component of a multimodal approach to postoperative pain management [10].

By decreasing opioid consumption, CEA allows neonates to achieve adequate respiratory function more quickly, supporting timely extubation [11,12]. Additionally, by lowering pain scores [11,12], CEA can reduce agitation and further decrease opioid administration [13], thereby lowering the risk of complications such as reintubation due to cardiorespiratory events (e.g., desaturation, apnea, bradycardia). These benefits highlight the importance of epidural analgesia in enhancing postoperative recovery for neonates and infants undergoing surgery.

The role of CEA in facilitating early extubation in neonates is not well understood [11,12]. In Enhanced Recovery After Surgery (ERAS) protocols for neonates, CEA is moderately recommended for its potential to reduce opioid use [14], but it is not explicitly highlighted for facilitating extubation. Additionally, the impact of surgical procedures and a history of pulmonary conditions on extubation timing when CEA is used remains unclear. In our observation, despite the use of CEA, opioids are still commonly administered in many Neonatal Intensive Care Units (NICUs), including ours. Morphine, whether administered as an infusion or a bolus, can complicate successful extubation due to its sedative effects and potential to cause respiratory depression and precipitating reintubation. Decreasing opioid administration while CEA is used for intubated infants is challenging due to difficulties in pain assessment and the potential side effects of morphine, such as dependence and tolerance [7]. As a result, the likelihood of early extubation decreases.

To investigate the relationship between epidural analgesia, extubation, and key clinical outcomes that could influence extubation and reintubation, we performed a retrospective descriptive study involving 100 infants admitted to NICU after undergoing invasive surgical procedures and receiving CEA for pain control.

The study’s main aim was to examine the timing of extubation in infants who received an epidural catheter. The secondary aim was to identify factors that may delay the extubation, including the intubated status before the surgical procedure, type of surgical procedure, history of lung conditions, and opioid administration. We focused on postoperative morphine infusion administration as a potential contributing factor. The third aim is to investigate the number of infants requiring reintubation and the reintubation timing, in addition to analyzing potential contributing factors, such as morphine administration, pain, and sedation scores before the reintubation.

## 2. Materials and Methods

### 2.1. Study Design

This retrospective review was conducted at UPMC Children’s Hospital of Pittsburgh (CHP) with approval from the University of Pittsburgh Institutional Review Board (STUDY22120083 on 18 July 2023). The study adhered to the Declaration of Helsinki for ethical research involving human subjects and was classified as a minimal risk due to its retrospective nature. The use of de-identified data allowed a waiver of informed consent. The institutional electronic medical records were reviewed for neonates admitted to the UPMC CHP Neonatal Intensive Care Unit who received epidural catheters for postoperative pain control between July 2018 and April 2024. Three of the 103 patients identified were excluded due to preexisting tracheostomies, resulting in a final cohort of 100 infants.

### 2.2. Data Collection

Data were collected retrospectively from electronic medical records and included the following:

(1) Demographics (gestational age at birth, sex, corrected gestational age and weight at the time of surgery, history of preexisting lung conditions);
(2) Anesthetic and surgical data (ASA physical status, details about the surgical procedures, anesthesia and surgery duration times, the time for the epidural catheter placement, the time required for the epidural catheter placement, epidural catheter type, insertion site, and catheter tip location, opioids administered intraoperatively, timing for extubation);
(3) Postoperative data (timing for extubation and re-intubation, pain and sedation scores for postoperative days (POD) 0–5, opioid type, mode of administration, concentration and amount, medication used for epidural analgesia, epidural infusion duration, and time for epidural catheter removal).

### 2.3. Patient Characteristics

The study cohort included infants whose gestational ages ranged from 22 weeks and 3 days to 41 weeks and 5 days. The corrected gestational ages at the time of surgery ranged from 31 weeks and 3 days to 54 weeks and 5 days. All infants with no contraindications for epidural analgesia who underwent invasive surgeries and received an epidural catheter for postoperative pain control as per surgeon and NICU provider requests were included. Exclusion criteria included patients with preexisting tracheostomies.

### 2.4. Surgical Procedures Performed

The study encompassed 100 surgical procedures, including ileostomy closures (32), exploratory laparotomies (12), bowel resections (12), jejunostomy closures (6), diaphragmatic hernia repairs (3), duodenal atresia repairs (8), TEF repairs (9), and omphalocele closures (2), and neuroblastoma resection, pheochromocytoma resection, LADD’s procedure, and Nissen fundoplication (1 case each).

### 2.5. Anesthetic and Epidural Catheter Placement Details

Anesthetic techniques were not standardized and were determined by the attending anesthesiologist. Due to the patient’s age and surgical complexity, all procedures utilized general anesthesia with endotracheal intubation. The anesthesiologist chose to extubate the infants after surgery, following discussions with the surgeons regarding specific procedures and considering the risk of early removal of endotracheal tubes. However, sometimes, the NICU providers were worried about pain management and requested that the infant not be extubated. The epidural catheter was placed before surgery started in 83 infants and at the end of surgery for 17 infants. The catheters were placed in the operating room,—using a caudal approach, with the catheter advanced at a desired thoracic level (ranging from T4 to T12) for 70 infants [10,15] and an intervertebral approach for 30 infants [16,17]. The ultrasound was used to facilitate the needle placement in the correct location and to confirm the catheter tip at the desired spine level. Four patients had two separate epidural placements. The epidural catheter was placed and managed by a pain team comprised of two fellows- and an anesthesiologist skilled in pediatric regional anesthesia. Intraoperative medications for pain control and sedation included fentanyl (78 infants), methadone (1 infant), acetaminophen (47 infants), dexmedetomidine (21 infants), and ropivacaine 0.1–0.2%.

### 2.6. Medication for Postoperative Pain Control

The neonatologists managed the postoperative pain and sedation plans, except for the local anesthetic infusion, which was ordered by the pain team. Epidural medications included ropivacaine (0.05%, 0.1%, or 0.2%) with or without clonidine (1 or 2 mcg/mL)—17 infants. The pain team managed the epidural infusion until the epidural catheter was removed. Postoperative medications included morphine (95 infants), fentanyl (9 infants), acetaminophen (83 infants), dexmedetomidine (3 infants), benzodiazepines such as lorazepam and midazolam (33 infants).

Morphine administration as boluses or continuous infusion was often the primary medication used for pain relief and sedation. Occasionally, fentanyl was used instead of morphine. None of the infants received ketorolac, and acetaminophen intravenously was administered for all the infants with no concerns for liver disease. Postoperative medication administration was documented in 24 h intervals until the end of the day when the epidural catheter was removed.

#### Pain and Sedation Scales

Postoperative pain and sedation were assessed using the Neonatal Pain, Agitation, and Sedation Scale (N-PASS) [18], a validated neonate tool for assessing pain/agitation and sedation in infants with postoperative pain [18,19,20]. The N-PASS evaluates five categories: crying/irritability, behavioral state, facial expression, extremity tone, and vital signs. Based on observation, pain/agitation scores range from 0 to 10, while sedation scores range from 0 to −10 and are assessed after stimulation [18]. The pain and sedation scores documented by the nurse for the first five postoperative days were recorded from the medical records.

### 2.7. Study Aims

The primary aim of this study was to investigate the timing of extubation in infants who received an epidural catheter. Specifically, we examined the percentage of infants extubated in the operating room versus those extubated daily in the NICU.

The secondary aim was to identify factors that may delay extubation. To achieve this, we compared infants who were extubated in the operating room with those who remained intubated, as well as those who were intubated before surgery with those who were not. We analyzed demographic data, including a history of preexisting lung conditions, surgical characteristics, and opioid administration across the entire cohort and within two subgroups: (1) infants extubated at the end of surgery versus those who remained intubated and (2) infants intubated before surgery versus those who were not.

Regarding opioid administration, we identified infants who received opioids both intraoperatively and postoperatively. Daily opioid administration via boluses and continuous infusion was tracked until the epidural catheter was removed. We compared these variables across the subgroups above and analyzed the duration of epidural analgesia and opioid administration. Total postoperative opioid administration was converted to intravenous morphine equivalents (MMEs) in mg/kg. We hypothesized that opioid infusion could delay extubation and increase the likelihood of reintubation. To test this hypothesis, we compared the number of infants receiving opioid infusion within the subgroups, as well as the total cumulative opioid amounts between those receiving morphine infusion and those who did not. As morphine was the most commonly used opioid in this cohort, it was the primary opioid discussed in our study results.

The third aim was to determine the number of infants requiring reintubation and the reintubation timing. We also analyzed potential contributing factors, such as morphine administration (boluses and infusion) and pain and sedation scores before and after reintubation. This analysis of pain and sedation scores provided insight into the effectiveness of epidural analgesia, particularly in the context of reintubation.

#### Statistical Analysis

Descriptive statistics were calculated using medians and interquartile ranges (IQR) for continuous data and counts and percentages for categorical data. Differences between treatment groups were tested on continuous data using Mann–Whitney U tests, and categorical differences were tested using Chi-squared tests and Fisher’s exact tests. Data were visualized using histograms and correlation matrices. Treatment groups comparing (1) infants extubated or not in the operating room, (2) infants not intubated versus intubated at presentation to the operating room for the surgical procedure, and (3) infants on postoperative morphine infusion vs. no morphine infusion were analyzed for differences. Missing values were removed from all denominators and statistical testing. *p*-Values < 0.05 were considered significant. R software (version 4.3.1, R Core Team, 2023) was used for data management and analysis.

## 3. Results

### 3.1. Demographics

The final analysis included 100 infants, 43 females and 57 males. Table 1 presents the patients’ demographics and the details of anesthesia, surgery, and epidural. Sixty-two patients had a preexisting pulmonary condition.

### 3.2. Extubation After Completion of Surgery

#### 3.2.1. Extubation in the Operating Room

Forty-five patients were extubated at the end of the surgery, and 55 remained intubated.

##### Daily Extubation of the Infants That Remained Intubated at the End of Surgery

**Figure 1:**
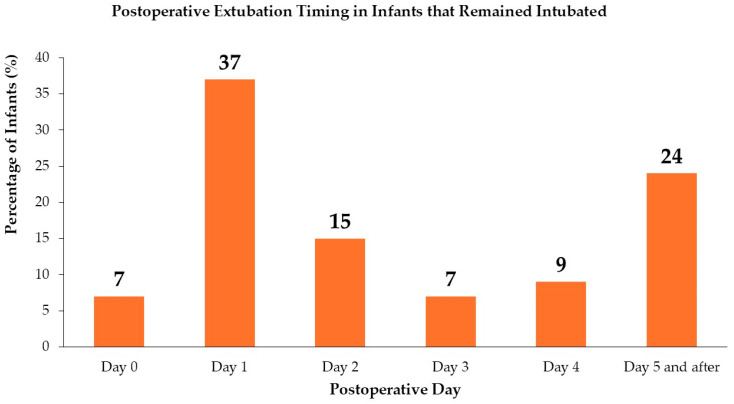
Daily extubation data for the patients who left the operating room intubated. The percentages (%) are reported daily.

The median/IQR extubation day for 55 intubated patients after leaving the operating room was 2 (1–4). Figure 1 presents the extubation days for this group.

##### Infants Extubated in the Operating Room vs. Infants That Remained Intubated

There is no difference in the demographics of the infants who remained intubated, except for a preexisting pulmonary condition (Table 2). A total of 32 out of 45 extubated infants received fentanyl intraoperatively, with a median (IQR) of 1.86 (0.89–2.90). A total of 46 out of 55 infants that remained intubated received fentanyl intraoperatively, with a median of 2.95 (1.66–4.05) (*p* < 0.001). Of the extubated infants, 16 required morphine infusion, while 17 of the intubated group required morphine, and 4 needed fentanyl infusions.

#### 3.2.2. Infants Intubated Before Surgery vs. Infants Not Intubated Before Surgeries

Eighty-eight patients were not intubated before surgery, and twelve patients were intubated.

##### Daily Extubation

**Figure 2:**
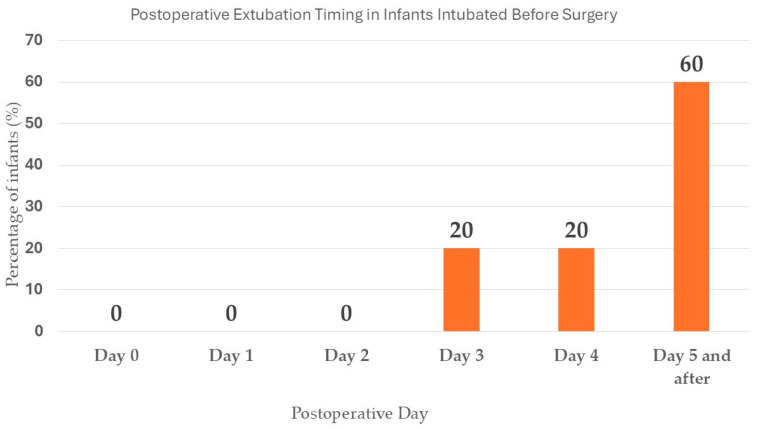
Daily extubation data for the patients who left the operating room intubated and were either not intubated or intubated before surgery. The percentages are reported daily. OR is the operating room. One patient was extubated on POD 5, 6, 16, 25, 29, and 37. One patient was never extubated.

Figure 2 presents how the infants intubated before surgery vs. those not intubated were extubated daily after leaving the operating room.

##### Infants Intubated vs. Infants Not Intubated Before Surgeries Characteristics

Thoracic procedures were significantly more common in patients intubated before surgery (42% vs. 9%, *p* = *0.029*). In total, 92% of intubated patients had a preexisting pulmonary condition, compared to 58% in the non-intubated group (*p = 0.027*). Opioid infusion administration was more common in intubated patients (58% vs. 34%) but not statistically significant (*p = 0.120*). The intubated infants received more morphine equivalents postoperatively [3.3 (0.8–8.3) vs. 0.5 (0.2–1.6) mg/kg, *p* = 0.008], but not intraoperatively (*p* = 0.069), or overall (*p* = 0.056).

### 3.3. Opioid Administration

#### 3.3.1. Intraoperative Opioid Administration

Twenty-two patients did not receive any opioids. Seventy-eight patients received fentanyl (2.49 [1.3–3.73] mcg/kg).

#### 3.3.2. Postoperative Opioid Administration

Three infants did not receive opioids postoperatively. The median/IQR of the POD until the patient received morphine boluses was 3 (3–3). The median/IQR of the POD until the patient received fentanyl boluses was 3 (2–4). The total median morphine equivalent administered was 0.53 (0.19–2.13) mg/kg.

##### Daily Opioid Administration

Table 3 presents the number of patients receiving daily opioid administration.

##### Opioid Continuous Infusion

Thirty-seven infants received opioid infusions (33 morphine, four fentanyl). The median/IQR of the POD until the patient received morphine infusion administration was 3 (3–3).

The infants who were on a morphine infusion received significantly more total morphine than those who only received bolus doses, totaling 2.7 (1.6–4.3) mg/kg vs. 0.3 (0.1–0.5) mg/kg (*p* < 0.001).

### 3.4. Reintubation Data

#### 3.4.1. Reintubation Rates

**Figure 3:**
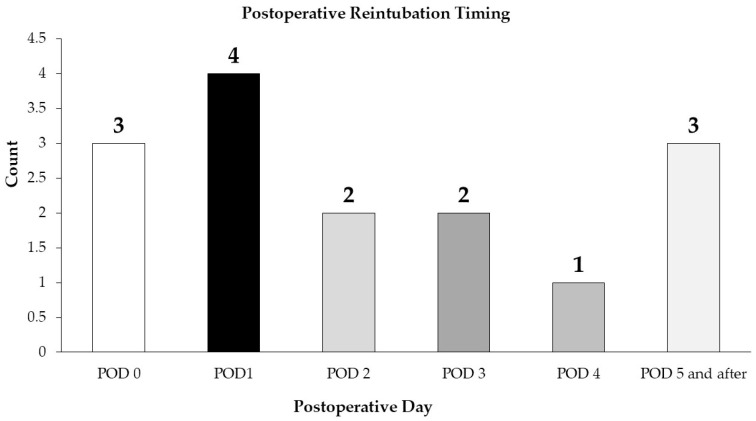
The postoperative day for reintubation. Counts represent the number of infants. POD is the postoperative day.

Fifteen patients required reintubation after extubation, thirteen of whom were not intubated before surgery, and two were intubated. The timing for reintubation is presented in Figure 3. One infant was extubated in the operating room but reintubated on POD 0 and re-extubated on POD 1. Three infants were reintubated on POD 5, 6, and 8.

#### 3.4.2. Opioid Administration and Reintubation

Fifteen infants required reintubation after extubation, and ten received morphine. These infants received a median of 5.5 (IQR: 3–8.75) boluses before reintubation and 7 (IQR: 2.25–11) boluses after reintubation. Additionally, eight infants received a continuous morphine infusion, while seven did not. Infants who received morphine infusion required a median of 9.5 (IQR: 6.5–17) boluses after reintubation. Infants without morphine infusion required a median of 2 (IQR: 1.8–4.2) boluses after reintubation.

#### 3.4.3. Pain Before Reintubation

##### Postoperative Pain Scores

The maximum pain scores were highest on POD 0–2, with a median (IQR) of 4 (1–5), decreasing over time to a median of 0 by POD 5 (Table S1). The average pain score started at a median of 0.82 (IQR 0.22–1.5) on POD 0 and gradually decreased, reaching 0 by POD 5, indicating minimal or no pain for most patients (Table S1).

##### Pain Scores Before Reintubation

**Figure 4:**
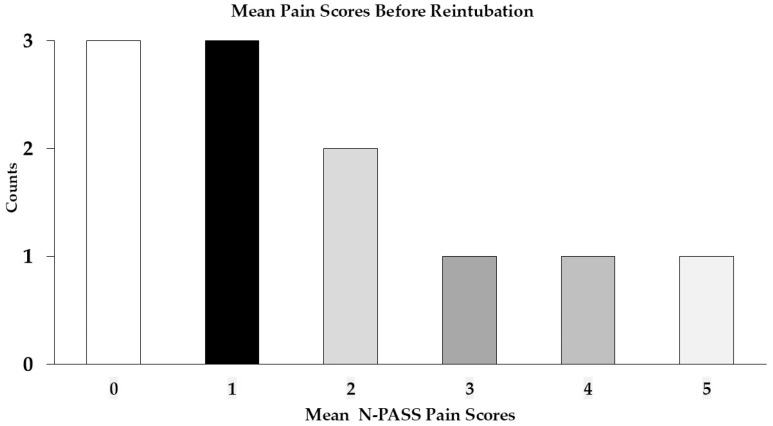
The median of three average pain scores before reintubation. Counts represent the number of infants. Postoperative pain scores were documented using Neonatal Pain, Agitation, and Sedation Scales (NPASS). A higher score indicates greater pain or agitation (the range of possible scores is 0 to 10).

The median of the average of three pain scores before reintubation was 1.67 (1–3) (Figure 4).

#### 3.4.4. Sedation Before Reintubation

##### Sedation Pain Scores

On POD 0, the minimum median sedation score was −3 (−5.75 to −1). By POD 1, it decreased to −2 (−4 to 0), and from POD 2 onward, sedation progressively improved, reaching a median score of 0 from POD 2 to POD 5. Average sedation scores decreased over time, from a median of −1.44 (−4.26 to −0.25) on POD 0 to 0 by POD 5 (Table S2).

**Figure 5:**
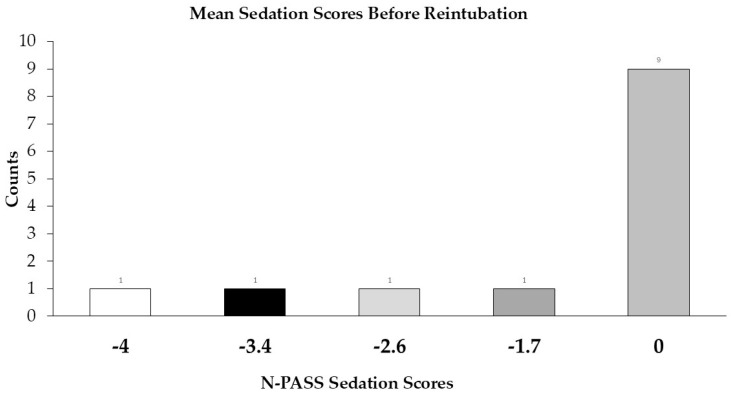
Mean sedation scores before reintubation. Counts represent the number of infants. Postoperative sedation scores were documented using Neonatal Pain, Agitation, and Sedation Scales (NPASS). A lower score indicates deeper sedation (the range of possible scores is –10 to 0).

The median of the average of three sedation scores before reintubation was 0 (−1.67–0) (Figure 5).

## 4. Discussion

In our study, we investigated how various factors, such as the type of surgical procedures, the history of pulmonary conditions, and opioid administration, contribute to the timing of extubation and the potential need for reintubation when epidural analgesia was used for pain control after surgery for 100 infants admitted in NICU.

Our findings showed that CEA when performed for infants who were not intubated preoperatively was associated with a 50% extubation success rate before leaving the operating room. Nevertheless, 56% of the 44 infants that remained intubated and were not intubated before surgery were extubated by the end of postoperative day 1. This extubation success rate suggests that the epidural provided adequate pain relief for these subjects.

Unfortunately, we did not have a control group to compare our extubation timing. We compared our results with the extubation rate provided by other researchers. Puthoff et al. reported an extubation success rate at the end of surgery of 10.5% for infants in whom an epidural catheter was not placed and 75% in 28 infants who underwent CEA [12]. This is a higher extubation rate than ours. However, their number of patients is smaller, and the infants studied are potentially different, as these authors excluded all emergent and urgent procedures and infants with weights less than 2 kg [12]. In contrast, as long the neuraxial procedure was not contraindicated, we placed epidural catheters in ill infants undergoing emergent surgery and less than 2 kg. It is not uncommon for these infants to remain intubated and require prolonged intubation, even when pain control is optimized with techniques such as epidural analgesia. In our study, we observed that nine infants who did not receive any fentanyl intraoperatively remained intubated after leaving the operating room.

To investigate potential factors influencing the extubation rate, we compared surgical procedures, infant characteristics, and opioid administration between the extubated patients and those that remained intubated at the end of surgery. The infants who were extubated in the operating room received less fentanyl during surgery compared to those who remained intubated. This finding suggests a potential benefit of CEA in reducing intraoperative opioid use and facilitating extubation at the end of surgery. A higher proportion of patients who remained intubated underwent thoracic procedures and had a preexisting pulmonary conditions. These factors could influence medical providers’ decisions to keep infants intubated after surgery.

We hypothesized that, after leaving the operating room, there would be a higher incidence of morphine infusion administration in infants who remained intubated compared to those who were extubated. Surprisingly, we found no significant difference in the percentage of infants requiring morphine infusion between these groups. This suggests that, for some infants, morphine administration did not cause delays in extubation when CEA was used for pain control.

Twenty-four percent of infants remained intubated after postoperative day 5, and one patient was never extubated and required a tracheostomy. This suggests that some infants had increased ventilation needs due to comorbidities and potential complications. Upon examining this subgroup, we found that the infants were highly premature (e.g., born at 23–24 weeks) and had associated comorbidities such as congenital diaphragmatic hernia, complex airway disease, chronic lung disease, pulmonary hypoplasia, and pulmonary overcirculation. These factors can impact the timing of extubation following invasive surgeries.

In our cohort, epidural catheter techniques were used for neonates who were already intubated upon arrival in the operating room. This practice has been debated due to perceptions that it offers limited benefits for these patients, as it is thought to neither facilitate extubation effectively nor significantly reduce opioid use. In our group, 50% of these neonates underwent thoracic procedures, and 92% had a preexisting lung condition. In total, 40% remained intubated until POD 5 or later. This suggests that preoperative intubation may indicate a higher risk for prolonged intubation.

We believe that CEA can aid in the extubation of infants who were intubated before surgery, even though, in our cohort, these infants required more postoperative morphine than non-intubated ones. For these infants, CEA helps by providing adequate pain control, which reduces agitation and improves hemodynamic and respiratory stability. This approach minimizes opioid dependence and tolerance, facilitates extubation, and lowers the risk of reintubation. Overall, incorporating CEA into postoperative care may reduce the reliance on systemic opioids in these infants.

Morphine is the gold standard for postoperative pain control and sedation for neonates [21]. It is administered as a continuous infusion or intermittent boluses [3,22]. Despite CEA being used in our cohort, the number of infants that required opioids remained high, the highest on POD 1 (83%), and remained high through POD 2 (78.4%). Despite these, most infants left intubated were extubated on POD 1. The opioid administration declined on POD 3 (62.7%), the median day for epidural catheter removal. Some infants still require opioids in later days (POD 4–5). This is concerning because neonatal metabolism—especially phase 2 metabolism—is underdeveloped compared to older children, and morphine is processed more slowly, leading to a more prolonged elimination half-life [23,24], delaying extubation and increasing the probability of reintubation.

Thirty-seven infants received continuous opioid infusion. It is unclear whether continuous opioid infusion reduces pain more effectively than intermittent boluses [3,22]. In our study, the infants on morphine infusion required higher doses of opioids. This may be due to opioid tolerance and dependence, which can develop quickly, particularly in preterm infants, with prolonged or continuous administration [7]. Surprisingly, some infants continued to receive morphine infusions even after extubation, without experiencing respiratory compromise, and some infants were successfully extubated while still receiving morphine infusions.

In our study, 15 patients required reintubation, and it was hypothesized that the primary reason for reintubation was uncontrolled pain and oversedation due to opioid administration by continuous infusion and additional boluses. However, several factors influence the risk of reintubation in neonates and infants following surgery. Inadequate pain control can lead to agitation, increased oxygen demand, and unstable hemodynamics (apnea, bradycardia), making it difficult for the neonate to maintain effective respiratory effort [13]. Prior opioid exposure can likely influence pain assessment and opioid administration and contribute to reintubation after surgery [25]. Residual sedation from prolonged or excessive opioid or sedative use may suppress respiratory drive, while underlying respiratory conditions, such as chronic lung disease or pulmonary hypertension, can further compromise breathing [26]. Surgical factors, particularly procedures involving the thoracic or abdominal regions, may impair diaphragmatic function or exacerbate postoperative pain, hindering effective respiratory efforts [27]. Hemodynamic instability, airway edema or obstruction, infections, or inflammatory states like pneumonia can also contribute to extubation failure.

We were unable to prove that our infants were reintubated due to uncontrolled pain, morphine administration, or resulting respiratory complications or oversedation; however, seven infants were reintubated by the end of POD 1. The pain and sedation scores before intubation were low, and no significant difference was observed between infants who received morphine infusions and those who did not in terms of the need for reintubation. In addition to the need for pain management requiring morphine infusion (2 infants), other reasons for reintubation were identified: acute respiratory failure, with or without apnea (10 infants), apnea secondary to pain medication and sedation (1 infant), complications developing during a subsequent procedure (1 infant) and failed extubation on POD 5 (1 infant).

We observed that most epidural catheters were removed on postoperative day 3, which aligns with the median duration for morphine boluses and infusion administration. These findings suggest that a structured protocol for epidural removal and opioid administration was followed. However, unlike pain management, the timing of extubation varied significantly, indicating that extubation was more dependent on the individual patient’s condition rather than solely on the analgesia provided by CEA and opioid administration.

Unfortunately, we were unable to demonstrate any influence of epidural analgesia or opioid administration on the timing of extubation. To further explore the relationship between CEA and potential factors that could affect extubation, we will discuss two neonates with weights under 2 kg who received continuous epidural analgesia.

The first patient was a 2-week-old infant born at 32 weeks of gestation, weighing 1.68 kg, with a ventricular septal defect, trisomy 21, and duodenal atresia, not intubated before surgery, scheduled for laparotomy. An epidural catheter was placed before surgery started and maintained for 4 days. The neonate received 0.6 mcg/kg of fentanyl during anesthesia. He was successfully extubated at the end of the surgery in the operating room. From POD 0 to POD 3, the infant received 0.18 mg/kg morphine. On POD 4, after the epidural catheter was removed, morphine consumption increased to 0.16 mg/kg. This case suggests that the epidural provided effective pain relief, supported early extubation, and reduced the need for systemic opioids.

The second patient was a 4-day-old premature infant born at 31 weeks of gestation, weighing 1.5 kg, with a congenital diaphragmatic hernia, intubated before surgery and scheduled for open repair. The neonate received 11 mcg/kg of fentanyl during anesthesia. An epidural catheter was placed at the end of surgery and maintained for 5 days following surgery. Despite the use of epidural analgesia, the infant required morphine infusions and additional boluses, totaling up to 1 mg/kg/day. The morphine was administered to provide adequate sedation and manage chest tube interventions. Pain scores remained very low throughout the recovery period. Despite daily increased opioid requirements, the neonate was successfully extubated on POD 4 and transitioned to bubble Continuous Positive Airway Pressure (CPAP). In this case, opioid consumption was high, and we cannot definitively show that the epidural decreased morphine consumption or facilitated extubation; however, the infant’s extubation occurred much earlier than expected, given the patient’s prematurity and the complexity of the surgical procedure. This case highlights the unique postoperative challenges faced by very preterm and low birth weight neonates, particularly in achieving adequate sedation while they are intubated.

### Study Limitations

This study has several limitations. As a retrospective descriptive analysis, it was impossible to assess cause-and-effect relationships directly. The absence of a control group makes it challenging to determine the effectiveness of continuous epidural analgesia. However, we compared our findings on opioid use and pain scores with existing data from the literature, and we observed consistency with previously reported outcomes. Puthoff et al. reported a median of 3.2 mg/kg MME with IQR of 1.2 to 7.4 in 21 infants that did not receive epidural analgesia and 0.77 (0.41–3.8) mg/kg MME in 30 infants that received epidural [12]. Our pain scores are lower than the pain scores reported in the groups that received or did not receive epidural analgesia (average N-PASS for the first 48 h in each patient < 4 in both groups) [12]. These support the assumption that epidural analgesia effectively manages postoperative pain.

Another limitation is the fact that we did not collect any information regarding the previous surgical procedures performed and patients’ prior opioid exposure—these factors, as noted by Peters et al. [11,25], could significantly influence postoperative opioid requirements and extubation outcomes. Lastly, some epidural catheters were placed at the end of the surgery and could affect the intraoperative opioid administration and the decision to extubate the infant at the end of the surgery. However, we did not see any relation between the timing of epidural placement and the percentage of infants extubated at the end of surgery. These limitations highlight the need for future research that incorporates prospective designs, control groups, and standardized assessment protocols to understand better and address the complexities of pain and opioid use in infant populations.

## 5. Conclusions

This study examined the relationship between preoperative intubation, pulmonary conditions, surgical procedures, opioid use, and postoperative extubation and reintubation in 100 infants who received CEA for pain management following significant surgeries. Our findings suggest that CEA is associated with earlier extubation in infants who were not intubated preoperatively, did not have a preexisting lung condition, and did not undergo a thoracic procedure. Lower postoperative opioid consumption was identified in infants who were not receiving a morphine infusion and were not intubated before surgery. The pain and sedation scores were low, demonstrating adequate pain and sedation in the postoperative period.

Notably, we found no direct correlation between opioid administration, pain and sedation scores, and reintubation events. However, the high proportion of infants requiring opioids, including infusions, suggests that further investigation is needed to understand these relationships fully. These results provide a framework for other institutions looking to implement epidural analgesia in the NICU to improve extubation and reintubation outcomes. Our findings contribute to the growing body of evidence supporting the integration of CEA into multimodal postoperative pain management for neonates and infants. However, further prospective studies are needed to identify the populations most likely to benefit from epidural analgesia and to develop strategies for minimizing opioid use in this vulnerable group.

## Figures and Tables

**Table 1:** The patient’s demographics and surgical, anesthesia, and epidural characteristics. Six patients had an ASA E (emergent) status. All the variables were reported as median and interquartile range (IQR). ASA is the American Society of Anesthesiology classification. POD is the postoperative day when the epidural is removed. Epidural catheter placement time represents the time required to place the epidural catheter.

Variable	Total Median (IQR) *N* = 100
Gestational age at birth (weeks)	34.5 (26.43–37.57)
Corrected gestational age at surgery (weeks)	40 (38.39–42.07)
Days of life at the surgery	55 (4.75–91.5)
Weeks of life at the surgery	7.86 (0.68–13.07)
Weight (kg)	3 (2.52–3.42)
ASA status	3 (3–3)
Epidural infusion duration (days)	2.88 (2.69–3.09)
POD of epidural removal	3 (3–3)
Anesthesia duration (minutes)	290.48 (248.13–351.43)
Surgery duration (hours)	2.74 (2.15–3.54)
Epidural catheter placement time (minutes)	23 (17–29.25)

**Table 2:** The extubation comparison table shows the patients who were left intubated versus extubated at the end of surgery. The extubation cohort is the denominator, *n*, and *N* represents the number of patients from each group—% shows column percentages. OR is the operating room. One patient was extubated in the operating room, reintubated on postoperative day 0, and extubated again on POD 1. Other procedures include laparoscopic-assisted colostomy (3) and laparoscopic treatment of intestinal malrotation.

Extubation Comparison Table Between Patients Extubated in the Operating Room and Remained Intubated					
	Variable Value	Total *N* = 100	Extubated in OR *N* = 45	Remained Intubated After OR *N* = 55	*p*-Value
			*n* (%)	*n* (%)	
Surgical Procedures	Thoracic Procedure	13 (13%)	0 (0%)	13 (24%)	0.002
	Thoracic Procedures and Circumcision	1 (1%)	0 (0%)	1 (2%)	
	Exploratory Laparotomy Bowel Resection	33 (33%)	15 (33%)	18 (33%)	
	Exploratory Laparotomy Bowel Resection and Circumcision	8 (8%)	6 (13%)	2 (4%)	
	Stoma Closure	29 (29%)	15 (33%)	14 (25%)	
	Stoma Closure and Circumcision	10 (10%)	5 (11%)	5 (9%)	
	Other	5 (5%)	4 (9%)	1 (2%)	
Preexisting Pulmonary Condition	Yes	62 (62%)	22 (49%)	40 (73%)	0.015
Received Opioid Infusion Postoperatively	Yes	37 (37%)	16 (36%)	21 (38%)	0.787

**Table 3:** Daily opioid administration while the epidural was maintained in situ. % shows percentages of the infants receiving fentanyl.

Opioid Administration While Patients Were Receiving Epidural Analgesia		
Postoperative Day	Patients with Epidural	Number of Patients (%)
0	100	80 (80%)
1	100	83 (83%)
2	97	76 (78.4%)
3	83	52 (62.7%)
4	19	14 (73.7%)
5	3	2 (66.7%)

## Data Availability

UPMC Children’s Hospital, Department of Anesthesia archived dataset analyzed.

## Supplementary Materials

The following supporting information can be downloaded at: https://www.mdpi.com/article/10.3390/children12030275/s1, Table S1: NPASS Pain Scores. Postoperative Daily Maximum and Average Pain Scores were documented using Neonatal Pain, Agitation, and Sedation Scales (NPASS). A higher score indicates greater pain or agitation (the range of possible scores is 0 to 10). One patient did not have documented pain scores on postoperative day 0; Table S2: NPASS Sedation Scores. Postoperative Daily Minimum and Average Sedation Scores were documented using Neonatal Pain, Agitation, and Sedation Scales (NPASS). A lower score indicates deeper sedation (the range of possible scores is –10 to 0). Two patients did not have documented pain scores on postoperative day 0.
