# Supplementary material for: Exploring the Interactions Between Epidural Analgesia, Extubation and Reintubation Outcomes in Infants in Neonatal Care Units: A Retrospective Cohort Study
Source: Children (Basel). 2025 Feb 24;12(3):275. doi: 10.3390/children12030275 (PMC11941318; doi:10.3390/children12030275)
Supplement: Supplementary file 1 [file children-12-00275-s001.zip › children-3474069-supplementary.pdf]

Supplemental Tables

Exploring the Interactions Between Epidural Analgesia, Extubation and Reintubation Outcomes in Infants in Neonatal Care Units: A Retrospective Cohort Study

Table S1. NPASS Pain Scores. Postoperative Daily Maximum and Average Pain Scores were documented using Neonatal Pain, Agitation, and Sedation Scales (NPASS). A higher score indicates greater pain or agitation (the range of possible scores is 0 to 10). One patient did not have documented pain scores on postoperative day 0.

NPASS Pain Scores	Number of patients	Postoperative Day	Total median (IQR)
Maximum Pain Scores	99	0	4 (1 - 5)
	100	1	4 (3 - 5)
	100	2	4 (2 - 5)
	100	3	3 (0 - 4)
	100	4	2 (0 - 4)
	100	5	0 (0 - 4)
Average Pain Scores	99	0	0.82 (0.22 - 1.5)
	100	1	0.54 (0.19 - 0.83)
	100	2	0.38 (0.12 - 0.68)
	100	3	0.17 (0 - 0.56)
	100	4	0.2 (0 - 0.82)
	100	5	0 (0 - 0.54)

Table S2. NPASS Sedation Scores**.** Postoperative Daily Minimum and Average Sedation Scores were documented using Neonatal Pain, Agitation, and Sedation Scales (NPASS). A lower score indicates deeper sedation (the range of possible scores is –10 to 0). Two patients did not have documented pain scores on postoperative day 0.

NPASS Sedation Scores	Number of patients	Postoperative Day	Total median (IQR)
Minimum Sedation Scores	98	0	-3 (-5.75 - -1)
	100	1	-2 (-4 - 0)
	100	2	0 (-2.25 - 0)
	100	3	0 (-1 - 0)
	100	4	0 (0 - 0)
	100	5	0 (0 - 0)
Average Sedation Scores	98	0	-1.44 (-4.26 - -0.25)
	100	1	-0.42 (-1.83 - 0)
	100	2	0 (-0.68 - 0)
	100	3	0 (-0.07 - 0)
	100	4	0 (0 - 0)
	100	5	0 (0 - 0)
